# Effectiveness of two‐drug therapy versus monotherapy as initial regimen in hypertension: A propensity score‐matched cohort study in the UK Clinical Practice Research Datalink

**DOI:** 10.1002/pds.4884

**Published:** 2019-09-03

**Authors:** Karine Marinier, Pauline Macouillard, Martine de Champvallins, Nicolas Deltour, Neil Poulter, Giuseppe Mancia

**Affiliations:** ^1^ Department of Pharmacoepidemiology and Real World Evidence Servier Suresnes France; ^2^ IT&M Stats Neuilly‐sur‐Seine France; ^3^ Center for Therapeutic Innovation in Cardiology Servier Suresnes France; ^4^ School of Public Health Imperial College London London UK; ^5^ University Milano‐Bicocca Milano Italy

**Keywords:** blood pressure (BP) control, comparative effectiveness, hypertension, initial regimen, monotherapy, pharmacoepidemiology, propensity score, two‐drug therapy

## Abstract

**Purpose:**

To compare the effectiveness on blood pressure (BP) of initial two‐drug therapy versus monotherapy in hypertensive patients.

**Methods:**

Using the Clinical Practice Research Datalink, linked with Hospital Episode Statistics and Office for National Statistics, we identified a cohort of adults with uncontrolled hypertension, initiating one or two antihypertensive drug classes between 2006 and 2014. New users of two drugs and monotherapy were matched 1:2 by propensity score. Main exposure was “as‐treated,” ie, until first regimen change. Primary and secondary endpoints were systolic and diastolic BP control and major adverse cardiovascular event (MACE), respectively. Hazard ratios (HR) and 95% confidence intervals (CI) were estimated using Cox proportional hazard models.

**Results:**

Of 54 523 eligible patients, 3256 (6.0%) were initiated to a two‐drug combination. Of these, 2807 were matched to 5614 monotherapy users. Mean exposure duration was 12.7 months, with 76.5% patients changing their initial regimen. Two‐drug therapy was associated with a clinically significant BP control increase in all hypertensive patients (HR = 1.17 [95%CI: 1.09‐1.26]), more so in patients with grade 2‐3 hypertension (HR = 1.28 [1.17‐1.41]). An increase of 27% in BP control (HR = 1.27 [1.08‐1.49]) was observed in patients initiating an ACEi+CCB combination compared with initiators of either single class. No significant association was found between two‐drug therapy and MACE. Several sensitivity analyses confirmed the main findings.

**Conclusions:**

Few patients initiated therapy with two drugs, reflecting UK guidelines' recommendation to start with monotherapy. This study supports the greater effectiveness of two‐drug therapy as the initial regimen for BP control.

**KEY POINTS**

- Except for high risk patients, monotherapy is still the usual initial therapy in hypertension. However, most patients require the combination of at least two drugs to achieve target blood pressure (BP) values.
- Using routinely collected health data, we assessed the comparative effectiveness of an initial regimen in hypertension based on two‐drug combination versus monotherapy.
- Our large population‐based cohort study supports the evidence of greater effectiveness of initiating two‐drug therapy over monotherapy for BP control, especially for patients with moderate or severe hypertension.
- These findings are in line with the greater emphasis on initial two‐drug therapy recommended by the new hypertension guidelines in Europe.

## 1 INTRODUCTION

Hypertension, defined as high blood pressure (BP) values ≥140/90 mmHg,^1^ is a common chronic condition, with an overall prevalence ranging from 20% to 45% in the general adult population and exceeding 70% for the elderly.^1^, ^2^, ^3^ The high burden of hypertension is related to significant cardiovascular morbidity and mortality caused by multiple outcomes, such as stroke, myocardial infarction, atrial fibrillation, and heart failure.^4^ Hypertension is also a major risk factor for chronic renal disease and for progression to end‐stage renal disease.^5^, ^6^

Beyond lifestyle changes, hypertensive patients are usually prescribed antihypertensive drugs to lower their BP and substantially reduce their cardiovascular risk. Several large meta‐analyses, using data from randomized clinical trials (RCTs) or observational studies, demonstrated that BP lowering significantly reduces vascular risk across various baseline BP levels and comorbidities.^5^, ^7^, ^8^, ^9^, ^10^ Pharmacological options for initiation and maintenance of antihypertensive therapy include diuretics, beta‐blockers (BB), calcium channel blockers (CCB), angiotensin‐converting enzyme inhibitors (ACEi), and angiotensin receptor blockers (ARB). Until recently, European and US guidelines mainly recommended starting therapy with a single drug but approved initiating combination therapy for patients at high risk or with markedly high BP.^1^, ^11^, ^12^ Conversely, the NICE guidance does not recommend this initial strategy even for severe hypertensive patients in the UK.^13^ Evidence suggests that monotherapy can effectively reduce BP in only a limited number of hypertensive patients, and most subjects require the combination of at least two drugs to achieve BP targets.^14^ A large meta‐analysis, which compared the effects of combining drugs versus doubling dose across 42 trials, concluded that combining therapies from different classes is approximately five times more effective in lowering BP than increasing the dose of one drug.^15^ Therefore, there is no debate about the effectiveness of the combination strategy, but the question is more whether it should always be preceded by monotherapy, or whether combination therapy may be the initial approach.^16^ RCTs tend to confirm that initial two‐drug combination strategies achieve earlier BP control when compared with strategies that add a second drug after monotherapy.^15^, ^16^, ^17^, ^18^, ^19^, ^20^, ^21^, ^22^, ^23^ Similar evidence has accumulated in observational studies to some extent.^24^, ^25^, ^26^, ^27^, ^28^ However, most studies were based on US data and limited to evaluate a BP outcome only in a short‐term period.^24^, ^26^, ^28^ Besides, most previous studies did not address bias due to confounding by indication, caused by differential prescribing between combination therapy and monotherapy. To overcome these limitations, we designed a large population‐based study in UK comparing new users after propensity score (PS) matching. The primary objective was to investigate the effectiveness of antihypertensive drugs initiated as a two‐drug combination versus monotherapy on BP control and cardiovascular risk. Secondary objectives were to assess whether the effects were similar according to the severity of hypertension, and in the patients initiating a combination of ACEi+CCB compared with those initiated with either single class.

## 2 METHODS

### 2.1 Study design

An observational retrospective cohort study using electronic medical records of new users of antihypertensive drug classes in the United Kingdom was conducted. Patients initiated on combination therapy were matched 1:2 with those starting monotherapy by using PS in order to minimize confounding by indication, with covariates in the PS measured at baseline to avoid adjustment for intermediate characteristics in the causal pathway.^29^, ^30^, ^31^

### 2.2 Data sources

Primary care data were obtained from the Clinical Practice Research Datalink (CPRD) GOLD, which contains computerized longitudinal medical records for ~15 million patients in the United Kingdom.^32^ Inpatient care data were provided by linkage to the Hospital Episode Statistics (HES) data warehouse which compiles hospitalization records from April 1997 onwards in England. Mortality data (dates and causes) were available from the Office for National Statistics (ONS) whilst data on socio‐economic status were obtained by proxy from the Indices of Multiple Deprivation (IMD).^33^ These linked data are available only for patients from English practices having consented to linkage, representing 60% of the CPRD GOLD population.

CPRD and linked datasets have proved to be valid data sources to investigate BP^34^ and cardiovascular diseases, with a positive predictive value above 90% for acute myocardial infarction identified in primary care or through hospital admission.^35^

### 2.3 Study population

The study population was formed from research quality acceptable patients in CPRD, who were eligible for HES and ONS data linkage. Adult male and female patients were included if they had received any prescription for ACEi, ARB, CCB, thiazide and thiazide‐like diuretics (TZD), and/or BB between 1 January 2006 and 31 December 2014, without any prescription of an antihypertensive drug in the previous 6 months. Treatment initiation was defined as the index date (ID). Only patients initiating a single drug or two‐drug therapy at the ID were considered. In addition, eligible patients should have at least 12 months of prior follow‐up in their practice for past medical history, evidence of hypertension (reported diagnosis code and/or repeated elevated BP measures, defined as systolic BP ≥140 mmHg and/or diastolic BP ≥90 mmHg) at/or before the ID, and an elevated BP measure (SBP ≥140 mmHg and/or DBP ≥90 mmHg) within 3 months before the ID. Patients were excluded if they had secondary hypertension or heart failure before the ID, or a stroke or myocardial infarction reported in the year preceding the ID.

Patients were followed from their ID until outcome occurrence, exposure end, transfer out, death, last collection date for the practice, or end of coverage in linked datasets (29 February 2016), whichever occurred first. Patients were only allowed to enter the study cohort once.

### 2.4 Exposure

Exposure was based on prescription records of general practitioners. Monotherapy included any single drug amongst ACEi, ARB, CCB, TZD, and BB. Combinations of two drugs referred to a single‐pill (fixed‐dose) combination or dual free combination amongst the following: ACEi and CCB, ACEi and TZD, ACEi and BB, ARB and CCB, ARB and TZD, ARB and BB, CCB and TZD, CCB and BB, and TZD and BB. In case of dual free combination, both drugs had to be prescribed on the same day at the ID for the patients to be considered in this group.

Given that the overall antihypertensive drug discontinuation is high^36^, ^37^, ^38^, the main exposure was defined “as‐treated,” ie, until first regimen change (if any), expressed as a change in the number of concomitant antihypertensive classes (add or remove ≥1 class), thus ignoring any dose change, within‐class drug change or class switch. Treatment episodes were first built by class (ACEi, ARB, CCB, TZD, and BB), with prescriptions assembled in episodes if they overlapped or if the gap between the end of a prescription and the next prescription start was <60 days. Thus, discontinuation of a class episode was defined as the absence of a refill prescription within the time frame of 60 days, generally corresponding to two missed prescriptions.^39^ Finally, concomitant exposure was valid if episodes of different antihypertensive classes overlapped for at least 30 days. If the concomitant period was shorter, it was considered as a class switch.

### 2.5 Outcomes

The primary endpoint, defined as the first occurrence of BP control (SBP <140 mmHg and DBP <90 mmHg), was identified from primary care data. The secondary outcome was the first occurrence of a major adverse cardiovascular event (MACE), defined as a composite of acute nonfatal myocardial infarction, nonfatal stroke, and cardiovascular death, identified by READ and/or ICD10 codes used in primary care, inpatient, or mortality data. Myocardial infarction and stroke were considered as nonfatal if no record of death was reported within 30 days after their reported date.

### 2.6 Statistical analyses

Primary analysis focused on the comparison of the two‐drug combination versus monotherapy. A PS based on a logistic regression model was used to estimate the probability of receiving two‐drug therapy versus monotherapy using all baseline covariates with empirical inclusion criteria for appropriate selection of variables.^40^ Confounders were identified from all investigated data sources and included demographics, lifestyle, vital signs, history of hypertension, medical history, comedications, lipid tests, renal function tests, other laboratory tests, health care utilization, and prior follow‐up duration (Appendix—Table S1). The whole available look‐back period was assessed for the medical history covariates, as well as their timing relative to the ID to better control for confounding.^41^

Each patient initiated with a two‐drug combination was matched to two patients initiated with monotherapy by PS with greedy matching without replacement, using calliper with a prespecified width of 0.2 of the standard deviation (SD) of the logit of the PS. Baseline characteristics of patients were described before and after PS matching. The weighted absolute standardized difference with a threshold of 0.1 was used to assess balance of covariates between treatment groups.^42^, ^43^

Incidence rates were estimated with 95% confidence intervals (CI) based on the Poisson or normal distribution. Endpoints were compared between two‐drug combination and monotherapy using Cox proportional hazard models with a robust variance estimator to account for the matched nature of the data. Hazard ratios (HR) were provided with their 95% CI. If unbalance remained for some covariates after matching, they were further adjusted in the Cox model.^44^ BP control after the ID, which represented an intermediate variable of the causal pathway from treatment to MACE, was considered as a time‐varying covariate for the MACE endpoint. Time to endpoint was plotted using Kaplan‐Meier survival analyses.

### 2.7 Subgroup and sensitivity analyses

Subgroup analyses were conducted: first, in patients with grade 1 hypertension (SBP in 140‐159 mmHg and/or DBP in 90‐99 mmHg) at the ID; second, in patients who received ACEi and CCB in combination versus ACEi or CCB as their initial single treatment. This latter subgroup was chosen because these drug classes are amongst the preferred options for starting antihypertensive therapy, as recommended by the UK NICE guidance.^13^ Post‐hoc analyses were also performed in a third subgroup of patients with grade 2‐3 hypertension at the ID (grade 2: SBP/DBP in 160‐179/100‐109 mmHg, grade 3: SBP/DBP ≥ 180‐110 mmHg). A specific PS was modelled for matching patients in each subgroup.

The robustness of our findings was assessed through several sensitivity analyses. First, in order to evaluate the impact of the regimen change definition, the “as‐treated” analysis was repeated whilst varying the discontinuation gap from 60 to 90 days and the minimum overlap between concomitant classes from 30 to 60 days. Second, we followed patients in an intention‐to‐treat (ITT) approach, where exposure remained as defined at the ID, regardless of any subsequent changes in therapy. Third, to minimize the potential new‐user misclassification, a sensitivity analysis was carried out with a washout period duration extended to 1 year before treatment initiation. Statistical analyses were performed using SAS/PC Software version 9.2.

## 3 RESULTS

**Figure 1:**
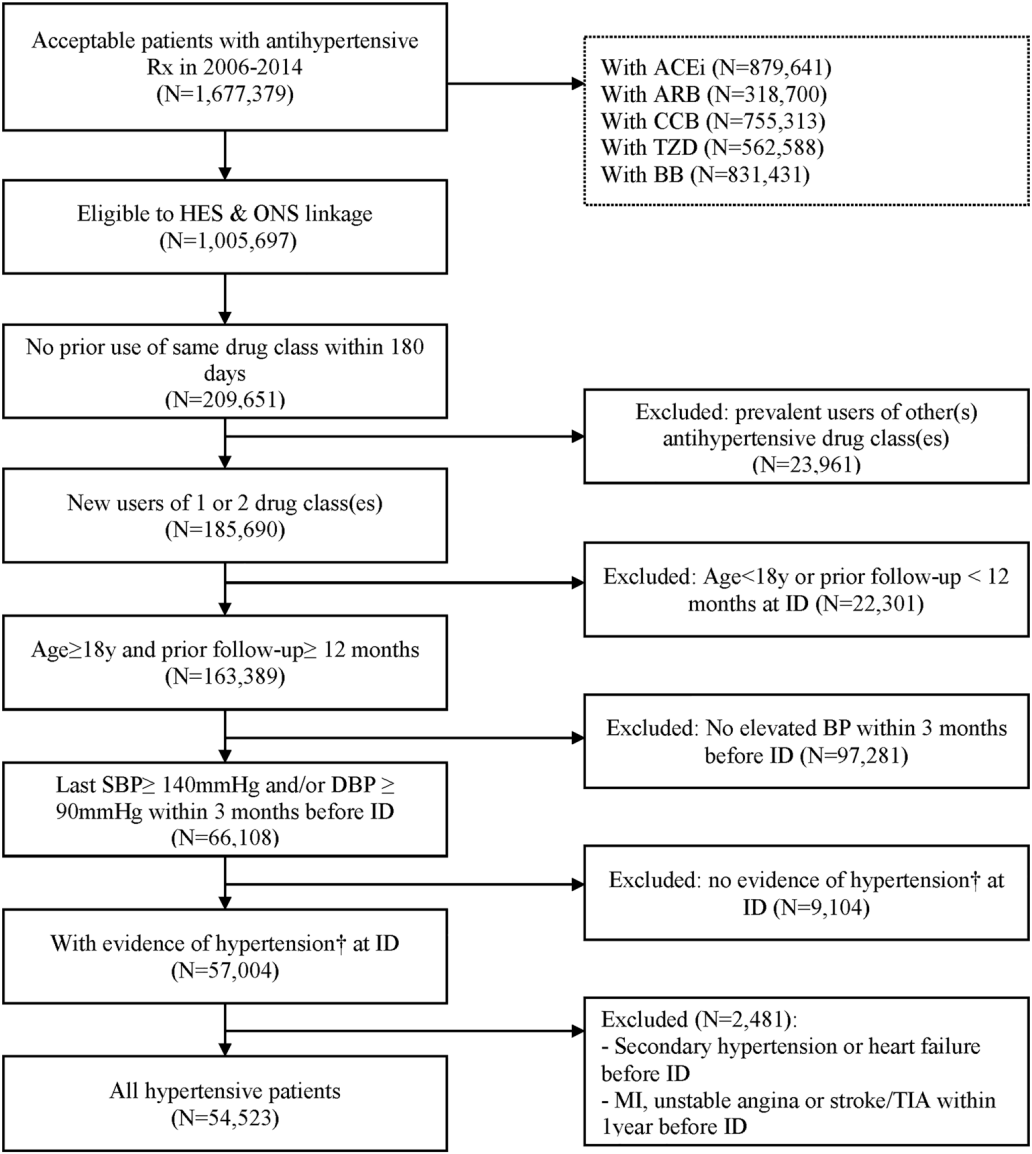
Patient flow chart. Abbreviations: ACEI, angiotensin‐converting enzyme inhibitors; ARB, angiotensin receptor blockers; BB, beta‐blockers; BP, blood pressure; CCB, calcium channel blockers; DBP, diastolic blood pressure; HES, Hospital Episode Statistics; ID, index date; MI, myocardial infarction; ONS, Office for National Statistics; Rx, prescription; SBP, systolic blood pressure; TIA, transient ischemic attack; TZD, thiazide and thiazide‐like diuretics.† reported diagnosis code before the index date and/or repeated elevated BP measures (defined as SBP ≥140 mmHg and/or DBP ≥90 mmHg) within a year before the index date.

**Figure 2:**
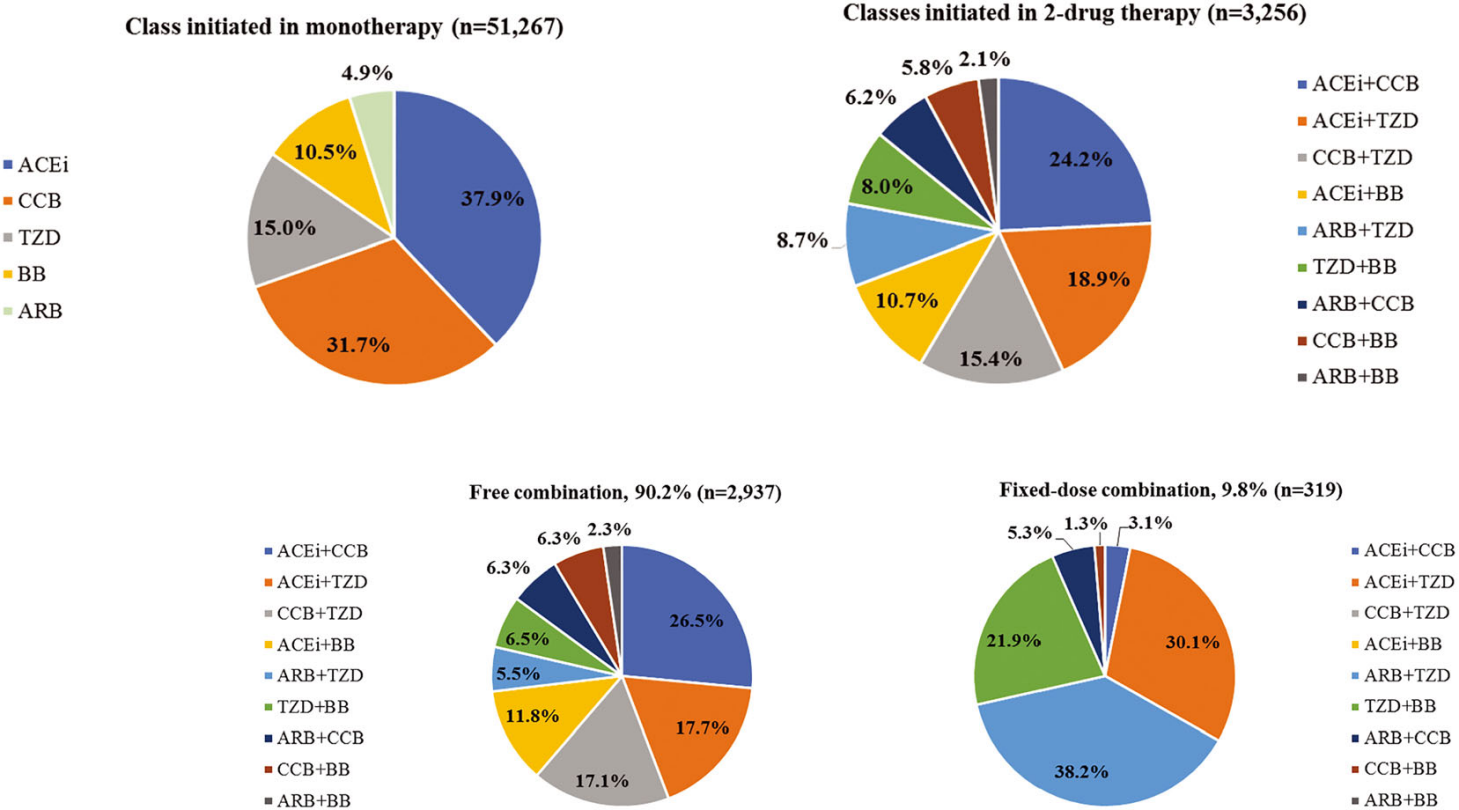
Prescription patterns of antihypertensive drug class (es) in the study population at treatment initiation. Abbreviations: ACEi, angiotensin‐converting enzyme inhibitors; ARB, angiotensin receptor blockers; BB, beta‐blockers; CCB, calcium channel blockers; TZD, thiazide and thiazide‐like diuretics. [Colour figure can be viewed at http://wileyonlinelibrary.com]

Of the 1 677 379 patients with an antihypertensive drug prescription identified in CPRD for the years 2006 to 2014, 185 690 were new users of one or two drug class (es). Finally, 54 523 were included in this study (Figure 1), with 51 267 (94.0%) initiated on monotherapy and 3256 (6.0%) started on two‐drug therapy. In this latter group, 90.2% and 9.8% had free and fixed‐dose combinations, respectively. ACEi and/or CCB were the most prescribed antihypertensive drugs (n = 36 469; 66.9%); 2.2% of included subjects (n = 787) received them concomitantly, which represented the most frequent combination of two antihypertensive drugs (Figure 2). Most patients (n = 36 529; 67.3%) had grade 2‐3 hypertension. Before matching, two‐drug new users were younger, more frequently men, and more likely to be current smokers than monotherapy users. They had a higher BMI and a lower socio‐economic status. Hypertension had been diagnosed for a longer period and was more severe (grade 3). Regarding their medical history, two‐drug users had a higher number of recent episodes of angina/ischemic heart disease and major coronary events. Before the 6‐month washout period used for the new‐user definition, they had received more prescriptions and classes of previous antihypertensive medication and stopped it more recently than monotherapy new users.

A total of 2807 combination users, representing 86.2% of all combination users, were matched to 5614 monotherapy patients. Baseline characteristics before and after matching are presented in Table 1. Matching restored balance on all confounders in all hypertensive patients (Appendix—Figure S1). In the subgroup with grade 1 hypertension, 923 (75.7%) combination users were matched to 1846 monotherapy users whilst 1737 (85.2%) and 3474 patients were matched in the subgroup with grade 2‐3 hypertension. A total of 778 (98.9%) new users of ACEi+CCB were compared with 1556 new users of ACEi or CCB alone.

**Table 1:** Main baseline characteristics of study population

	Full Cohort Before Matching			Matched Cohort		
	Monotherapy	Two‐Drug Therapy	ASD	Monotherapy	Two‐Drug Therapy	ASD
	N = 51 267	N = 3256		N = 5614	N = 2807	
*Demographic and lifestyle*						
Men (%)	44.4	52.4	0.16	51.3	51.6	0.01
Mean age in years, (SD)	62.0 (13.9)	60.3 (14.0)	0.13	60.8 (14.5)	60.7 (14.1)	0.02
Ethnicity (%)						
Missing	16.5	21.5		20.5	20.9	
White	93.3	87.0	0.21	87.9	88.5	0.02
Current smoking (%)	18.3	21.8	0.09	21.0	21.2	0.00
Mean BMI, kg/m^2^ (SD)	28.99 (5.72)	29.89 (6.05)	0.15	29.68 (5.92)	29.68 (5.97)	0.00
*History of hypertension*						
Mean SBP, mmHg (SD)	163.8 (18.1)	164.7 (21.5)	0.05	164.4 (20.3)	165.0 (21.2)	0.03
Mean DBP, mmHg (SD)	94.1 (11.8)	94.9 (14.2)	0.06	94.7 (13.1)	94.9 (14.1)	0.01
HT severity (%)						
Grade 1	32.7	37.5	0.1	36.8	36.0	0.02
Grade 2	42.4	30.9	0.24	32.8	32.4	0.01
Grade 3	24.9	31.7	0.15	30.5	31.6	0.03
Mean time in months since first HT diagnosis, (SD)	34.7 (45.7)	53.5 (45.6)	0.41	52.3 (47.0)	51.3 (45.7)	0.03
*Medical history (%)*						
Major coronary events	1.9	3.7	0.11	3.4	3.1	0.02
Angina/IHD	7.1	10.2	0.11	10.2	9.7	0.02
Stroke/TIA	3.6	3.7	0.00	3.9	3.7	0.01
Atrial fibrillation	3.3	2.9	0.02	3.3	3.1	0.01
Diabetes	11.0	11.2	0.01	10.2	11.0	0.03
PAD	1.9	1.7	0.02	1.8	1.8	0.00
Severe CKD	11.5	9.1	0.07	9.3	9.8	0.02
*AntiHT previous medication (>6 mo before ID)*						
AntiHT Rx (%)	73.0	92.5	0.54	91.8	91.3	0.02
Mean time since last antiHT Rx in months, (SD)	42.6 (45.6)	16.4 (23.2)	0.72	18.2 (24.5)	17.7 (24.9)	0.02
Mean number of antiHT classes (SD)	1.1 (1.0)	2.3 (1.1)	1.02	2.1 (1.3)	2.1 (1.1)	0.00
*Comedication in the year before ID (%)*						
Treatment of hyperglycaemia	7.1	6.6	0.02	5.9	6.3	0.02
Lipid‐lowering agents	21.0	25.4	0.10	23.8	23.8	0.00
Antiplatelets therapy	12.8	15.3	0.07	15.0	14.7	0.01
*Biological tests in the year before ID*						
Mean total cholesterol, mmol/L (SD)	5.48 (0.90)	5.60 (2.83)	0.06	5.53 (0.85)	5.54 (0.76)	0.02
Mean triglycerides, mmol/L (SD)	1.72 (1.23)	2.00 (3.65)	0.10	1.81 (1.19)	1.87 (0.65)	0.09
Mean Ratio HDL/LDL (SD)	0.50 (0.18)	0.47 (0.14)	0.15	0.48 (0.11)	0.47 (0.14)	0.03
*Use of health care resources in the year before ID*						
Mean number of GP visits (SD)	8.1 (7.2)	5.8 (6.8)	0.34	5.9 (6.1)	6.2 (7.1)	0.04
Mean number of hospitalization days (SD)	1.7 (9.2)	2.0 (11.6)	0.03	2.3 (12.5)	2.1 (12.2)	0.02

Abbreviations: ASD, absolute standardized difference; BMI, body mass index; BP, blood pressure; CKD, chronic kidney disease; DBP, diastolic blood pressure; GP, general practitioners; HDL, high‐density lipoprotein; HT, hypertension; ID, index date; IHD, ischemic heart disease; LDL, low‐density lipoprotein; PAD, peripheral arterial disease; Rx, prescription; SBP, systolic blood pressure; SD, standard deviation; TIA, transient ischemic attack.

In the matched cohort, the mean exposure duration until a potential regimen change was 12.7 months (SD: 19.4 months). Amongst monotherapy initiators, 75.5% changed their regimen afterwards, whilst 78.5% of those starting with a two‐drug combination added ≥1 class, switched to monotherapy or stopped therapy (Table 2).

**Table 2:** Exposure and regimen change in all hypertensive patients

	Matched cohort	
	Monotherapy	Two‐drug therapy
	N = 5614	N = 2807
*Exposure duration (months)*		
Mean (SD)	13.0 (19.9)	12.1 (18.5)
Median (IQR)	4.3 (1.8‐15.1)	4.1 (1.8‐13.7)
*No regimen change (%)*	24.5	21.5
Class persistence	14.7	14.5
Class switch	9.8	7.1
*Regimen change (%)*	75.5	78.5
Add ≥1 class	27.4	14.7
Remove ≥1 class	48.0	63.7
incl. therapy full discontinuation	48.0	46.7
incl. switch to monotherapy	‐	17.0
*Time to first regimen change (months)*		
Mean (SD)	7.7 (12.2)	7.9 (12.0)
Median (IQR)	2.8 (1.3‐8.2)	3.1 (1.8‐8.0)

Abbreviations: IQR, interquartile range; SD, standard deviation.

**Figure 3:**
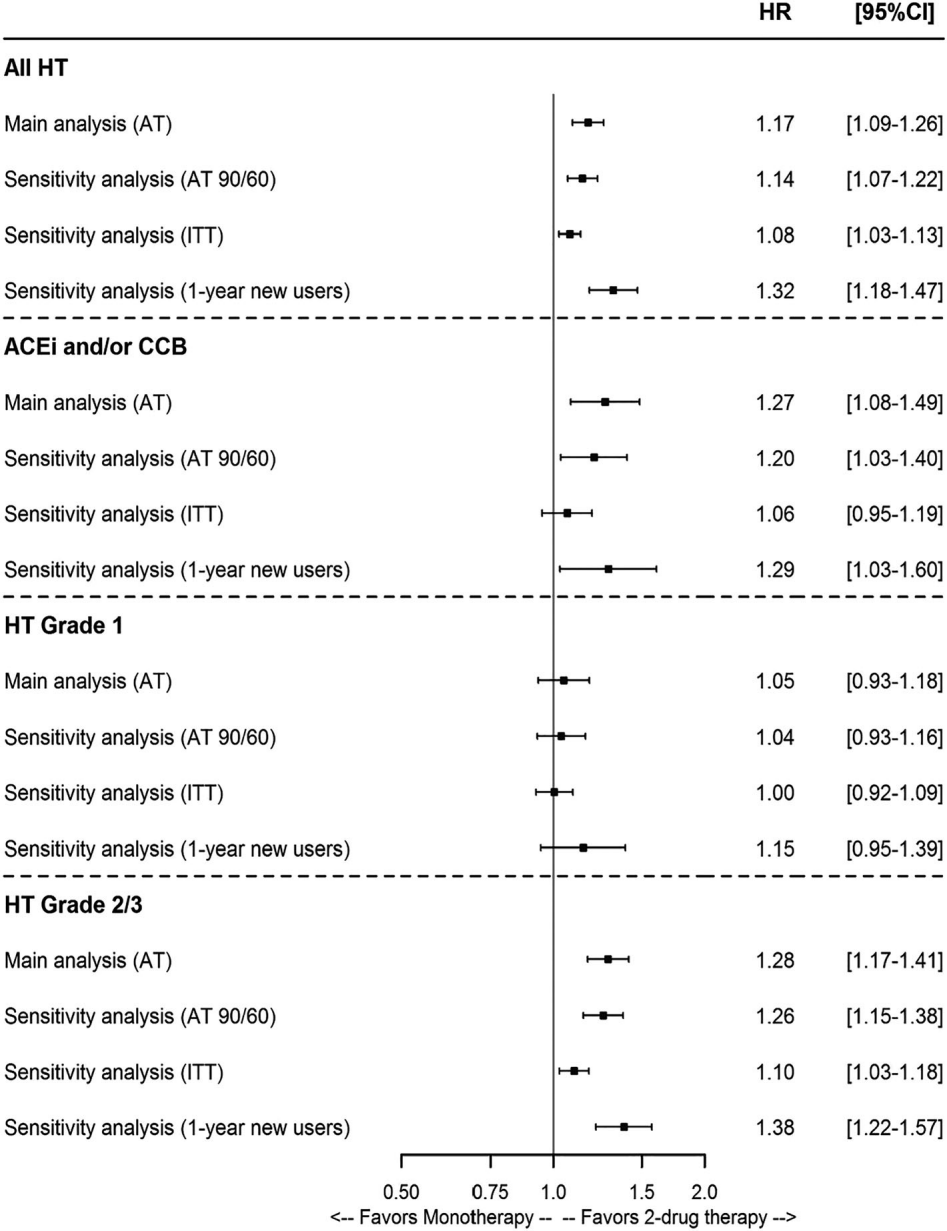
Forest plots for blood pressure control. Abbreviations: ACEI, angiotensin‐converting enzyme inhibitors; AT, as treated; CCB, calcium channel blockers; HT, hypertension; ITT, intention‐to‐treat.

Two‐drug therapy was associated with a clinically significant 17% BP control increase in the overall cohort (HR = 1.17 [95%CI: 1.09‐1.26]). In subgroups analyses, no association between two‐drug therapy and BP control was observed for patients with grade 1 hypertension, whilst the association was found higher in patients initiating a combination of ACEi+CCB (HR = 1.27 [1.08‐1.49]) and in patients with grade 2‐3 hypertension (HR = 1.28 [1.17‐1.41]) (Table 3 and Figure 3). Kaplan‐Meier survival curves for time to achieve BP control are available in Appendix—Figure S3.

**Table 3:** Incidence rates and hazard ratios for primary and secondary endpoints

		BP Control				MACE			
	Patients	Events	IR per 100 patient‐months	HR	[95%CI]	Events	IR per 1,000 patient‐years	HR	[95%CI]
All hypertensive patients									
Monotherapy	5614	1990	6.66			74	12.24		
Two‐drug therapy	2807	1134	7.90	1.17	[1.09‐1.26]	35	12.46	1.01	[0.68‐1.50]
Patients with ACEi and/or CCB									
Monotherapy	1556	557	6.68			19	11.73		
Two‐drug therapy	778	303	7.68	1.27	[1.08‐1.49]	8	10.54	0.81	[0.32‐2.06]
Patients with grade 1 hypertension									
Monotherapy	1846	794	7.63			22	9.37		
Two‐drug therapy	923	395	8.05	1.05	[0.93‐1.18]	13	13.48	1.44	[0.72‐2.87]
Patients with grade 2‐3 hypertension									
Monotherapy	3474	1122	6.25			56	15.75		
Two‐drug therapy	1737	693	8.12	1.28	[1.17‐1.41]	19	11.20	0.70	[0.41‐1.18]

Abbreviations: ACEi, angiotensin‐converting enzyme inhibitors; BP, blood pressure; CCB, calcium channel blockers; CI, confidence interval; HR, hazard ratio; IR, incidence rate; MACE, major adverse cardiovascular event.

Sensitivity analyses confirmed our initial findings. Considering alternative parameters of regimen change produced very similar results in the main population and the subgroups, whilst ITT results tended to attenuate the association between two‐drug therapy and BP control in all comparisons. When restricting the population to 1‐year new users, the association between two‐drug therapy and BP control was higher: HR = 1.32 [1.18‐1.47] in the overall cohort, 1.29 [1.03‐1.60] in patients with ACEi+CCB, and 1.38 [1.22‐1.57] in patients with grade 2‐3 hypertension.

No significant association was found between initial two‐drug therapy and MACE. Although nonstatistically significant, a positive trend was observed between two‐drug therapy and MACE for patients with grade 2‐3 hypertension (HR = 0.70 [0.41‐1.18]).

## 4 DISCUSSION

This large population‐based cohort study showed that an initial regimen with two‐drug therapy was associated with a clinically significant 17% increased BP control in hypertensive patients and was more beneficial (28% BP control increase) in patients with grade 2‐3 hypertension, when compared with monotherapy. Moreover, our findings were confirmed across all sensitivity analyses, with benefits as high as 32% and 38% for the 1‐year new users in all hypertensive and in patients with grade 2‐3 hypertension, respectively.

Prescription patterns for initiation of antihypertensive therapy in the United Kingdom were found to be largely consistent with the latest British guidelines which recommend initial monotherapy in all patients.^13^ In our study population, only 6.0% received two‐drug therapy as an initial regimen. ACEi and CCB were the most prescribed antihypertensive drugs alone or in combination. In patients initiating ACEi+CCB in combination, an increase of 27% in BP control was achieved.

To our knowledge, our study is the first population‐based study including a survival time‐to‐event analysis comparing monotherapy initiators versus two‐drug combination new users in hypertension, whilst controlling for indication bias. In particular, one of the main strengths of our study is the ability to control indication bias that occurs in nonrandomized observational comparative effectiveness studies. As confirmed by the baseline characteristics before matching, patients who start antihypertensive two‐drug therapy had more severe hypertension with a higher cardiovascular risk and/or more comorbidities. This difference in patient profiles results in a channelling bias due to confounding by indication when compared with patients initiated on monotherapy. To address the main biases, our study included only new users, PS matching, and an adjustment of unbalanced covariates (if any) in the Cox proportional hazard models. Double adjustment for the unbalanced covariates was indeed found to be the most robust method for removing residual confounding after PS matching.^44^

Our results are consistent with several previously published studies.^24^, ^25^, ^26^, ^27^, ^28^ However, some studies comparing two‐drug therapy initiation versus delayed drug combination were therefore subject to a selection bias towards patients for which monotherapy had failed. Confounding by indication induced by selective differential prescribing was also present in most previous studies. A recent retrospective cohort which included 48 131 patients with hypertension diagnosed between 2008 and 2010 from The Health Improvement Network (THIN) UK general practice medical records database provided findings of similar magnitude to ours.^45^ In this study, the vast majority (95.8%) of patients were also initiated on monotherapy. Starting on combination therapy was found to increase the odds of achieving BP control relative to starting with monotherapy in patients with high cardiovascular risk (OR: 1.23; 95% CI: 1.06 to 1.42). However, this study was limited to a 6‐month follow‐up, did not investigate any cardiovascular endpoint, and did not address indication bias.

Based on large, longitudinal, and population‐based data sources, our study covered a long‐time period (Jan 2006‐Feb 2016). CPRD linked data sets were found to provide reliable medical data and an accurate picture of the patient journey through primary care, hospitalization, and mortality.^33^ Moreover, outcomes of interest as well as diagnostic codes used in this study were already validated across CPRD and linked databases.^34^, ^35^ The association between BP and the occurrence of cardiovascular diseases, such as myocardial infarction, stroke, and heart failure, was also confirmed when using linked data of CPRD, HES, and mortality statistics.^8^

Nevertheless, several limitations should be acknowledged. The main limitation is that the new‐user definition was based on a washout period of 180 days which does not equate to a true incident new‐user design, as patients were allowed to have received prescriptions for antihypertensive drugs in the preceding period. Because this introduced a mix of truly new users and users restarting therapy after at least 6 months without treatment, we carefully defined several covariates to quantify this prior use and introduced them in the PS modelling in order to balance monotherapy and two‐drug therapy users on these covariates after PS matching. These covariates were highly unbalanced between groups before matching and well balanced after matching (see Appendix). We believe that this approach is a reasonable attempt to minimize the bias due to the mix of truly new and “restarting” users and enhances the validity of our results. Second, as expected and according to current guidelines in the United Kingdom, few patients were initiated on two‐drug therapy during the study period. This decreased the statistical power of comparison for the secondary MACE outcome, for which no definite conclusion was reached despite the slightly positive trend in favour of two‐drug combination for patients with grade 2‐3 hypertension. With the as‐treated exposure to a specific regimen not exceeding 13 months on average, this period was long enough to observe a protective effect of initial two‐drug therapy on BP but may be too short for a significant reduction in MACE. Third, study findings may not be generalizable to all patients on monotherapy, as PS‐matched analyses made the reference group similar to combination initiators. Another limitation concerns adherence which might be overestimated as drug prescribing does not reflect the true drug use. Nevertheless, adherence was reported to be lower in patients with free combination therapy,^46^ and this may have led to an underestimation of the true effectiveness of initial combination strategy in our study. Indeed, two recent studies showed a significant lower risk of serious cardiovascular events associated with fixed‐dose combination therapy, either versus multipill therapy^47^ or versus monotherapy.^48^ Lastly, PS matching can only control for measured confounding and residual confounding due to unmeasured covariates cannot be ruled out, even though our PS models included almost 50 covariates. To our knowledge, the key confounders were measured in our study. In that context, it could be assumed that remaining unmeasured confounders are possibly associated to already included covariates and would have therefore a limited impact on our conclusions.

In summary, this population‐based observational study supports the evidence of greater effectiveness of initiating two‐drug therapy over monotherapy for BP control, especially for moderate and severe hypertensive patients. This finding echoes the greater emphasis on initial two‐drug therapy in the recent update of the clinical guidelines in Europe which recommend treatment initiation with two drugs, preferably in a single pill form, in most patients with a BP ≥140/90 mmHg.^49^

## ETHICS STATEMENT

This study was approved by the Medicines and Healthcare products Regulatory Agency Independent Scientific Advisory Committee (protocol number 16_166R), and the protocol was made available to the journal reviewers.

## CONFLICT OF INTEREST

K.M., N.D., and M.d.C. are employees of Servier, a pharmaceutical company which manufactures blood‐pressure lowering agents. P.M. is an employee of IT&M Stats and currently works as a Servier contractor. N.P. has received financial support from several pharmaceutical companies which manufacture blood‐pressure lowering agents, for consultancy fees, research projects and staff, and for arranging and speaking at educational meetings. He holds no stocks and shares in any such companies. G.M. has received speaking fees from several companies (Servier, Menarini, Ferrer, Recordati, Merck Serono, Amgen, Medtronic, Fukuda), has been a member of advisory boards (Ferrer, Menarini, Sanofi). He holds no stocks or has no any other business participation in any such companies.

## Supporting information

Data S1. Supporting information
