# Supplementary material for: Effectiveness of two‐drug therapy versus monotherapy as initial regimen in hypertension: A propensity score‐matched cohort study in the UK Clinical Practice Research Datalink
Source: Pharmacoepidemiol Drug Saf. 2019 Sep 3;28(12):1572–82. doi: 10.1002/pds.4884 (PMC6916605; doi:10.1002/pds.4884)
Supplement: Supplementary file 1 — Data S1. Supporting information [file PDS-28-1572-s001.docx]

**APPENDIX**

[Table 1: Description of covariates used in the propensity score 2](#_Toc13763782)

[Figure 1a: Absolute standardized differences of covariates before and after propensity score matching in all hypertensive patients 5](#_Toc13763783)

[Figure 1b: Absolute standardized differences of covariates before and after propensity score matching in patients with ACEi and/or CCB 6](#_Toc13763784)

[Figure 1c: Absolute standardized differences of covariates before and after propensity score matching in patients with grade 1 hypertension 7](#_Toc13763785)

[Figure 1d: Absolute standardized differences of covariates before and after propensity score matching in patients with grade2-3 hypertension 8](#_Toc13763786)

[Figure 2: Distribution of propensity score before and after matching 10](#_Toc13763787)

[Figure 3: Kaplan Meier survival curves for time to achieve blood pressure control 11](#_Toc13763788)

# Table 1: Description of covariates used in the propensity score

| **Category** | **Name of covariate** | **Type of covariate and timepoint** | **Modalities** |
| --- | --- | --- | --- |
| **Demographics** | Year of inclusion | Qualitative, time-fixed at ID | 2006, 2007, 2008, 2009, 2010, 2011, 2012, 2013, 2014 |
|  | Gender | Qualitative, time-fixed at ID | Male, female |
|  | Age (years) | Qualitative, time-fixed at ID | 18-54, 55-64, 65-74, ≥75 |
|  | Ethnicity | Qualitative, time-fixed at ID | White, Asian, African, mixed, other ethnic group |
|  | Patient IMD (quintiles) | Qualitative, time-fixed at ID | - |
|  | Region | Qualitative, time-fixed at ID | North East, North West, Yorkshire & The Humber, East Midlands, West Midlands, East of England, South West, South Central, London, South East Coast |
| **Lifestyle** | Smoking status | Qualitative, time-fixed, most recent record at/before ID | Never, current, past |
|  | Heavy alcohol use | Qualitative, time-fixed, at or in the year before ID | Yes, no |
| **Vitals signs** | BMI (kg/m2) | Continuous, time-fixed, most recent record at/before ID | - |
| **History of hypertension** | Systolic blood pressure (mmHg) | Continuous, time-fixed, most recent record at/90days before ID | - |
|  | Diastolic blood pressure (mmHg) | Continuous, time-fixed, most recent record at/90days before ID | - |
|  | Severity of HT | Qualitative, time-fixed, most recent record at/90days before ID | Grade 1, 2 or 3 |
|  | Isolated systolic HT | Qualitative, time-fixed, most recent record at/90days before ID | Yes, no |
|  | Isolated diastolic HT | Qualitative, time-fixed, most recent record at/90days before ID | Yes, no |
|  | Time since first diagnosis of HT (years) | Qualitative, time-fixed, anytime before ID | No, 0-1, 2-5, >5 |
|  | Family history of HT | Qualitative, time-fixed at/before ID | Yes, no |
|  | Repeated elevated BP measures | Qualitative, time-fixed at 90 days before ID | Yes, no |
|  | Number of BP readings | Continuous, time-fixed at/in the year before ID | - |
| **Medical history** | Time since last diagnosis of coronary major events (years) | Qualitative, time-fixed at/before ID | No, 1-5, >5 |
|  | Time since first diagnosis of angina/IHD (years) | Qualitative, time-fixed at/before ID | No, 0-1, 2-5, >5 |
|  | Family history of IHD | Qualitative, time-fixed at/before ID | Yes, no |
|  | Time since last diagnosis of stroke/TIA (years) | Qualitative, time-fixed at/before ID | No, 1-5, >5 |
|  | Family history of stroke/TIA | Qualitative, time-fixed at/before ID | Yes, no |
|  | Time since first diagnosis of atrial fibrillation (years) | Qualitative, time-fixed at/before ID | No, 0-1, 2-5, >5 |
|  | Time since first diagnosis of diabetes (years) | Qualitative, time-fixed at/before ID | No, 0-1, 2-5, >5 |
|  | Family history of diabetes | Qualitative, time-fixed at/before ID | Yes, no |
|  | Time since first diagnosis of PAD (years) | Qualitative, time-fixed at/before ID | No, 0-1, 2-5, >5 |
| **Comedications** | Anti-hyperglycemic drugs | Qualitative, time-fixed in the year before ID | Yes, no |
|  | Lipid-lowering agents | Qualitative, time-fixed in the year before ID | Yes, no |
|  | Antiplatelet therapy | Qualitative, time-fixed in the year before ID | Yes, no |
|  | Time since last HT prescription (months) | Qualitative, time-fixed > 180 days before ID | None, 6-12, 13-24, >24 |
|  | Nb of anti-HT classes previously used | Continuous, time-fixed >180 days before ID | - |
| **Polymedication** | Prescriptions in at least 3 different pharmacological classes | Qualitative, time-fixed in the year before ID (ID excluded) | Yes, no |
| **Laboratory tests** | At least one lipid test | Qualitative, time-fixed in the year before ID | Yes, no |
|  | Total cholesterol (mmol/L) | Continuous, time-fixed in the year before ID | - |
|  | Triglycerides (mmol/L) | Continuous, time-fixed in the year before ID | - |
|  | HDL (mmol/L) | Continuous, time-fixed in the year before ID | - |
|  | LDL (mmol/L) | Continuous, time-fixed in the year before ID | - |
|  | Ratio Cholesterol/HDL | Quantitative, time-fixed in the year before ID | - |
|  | Ratio HDL/LDL | Quantitative, time-fixed in the year before ID | - |
|  | At least one renal test | Qualitative, time-fixed in the year before ID | Yes, no |
|  | Classification of CKD using GFR category (mL/min/1.73m²) | Qualitative, time-fixed in the year before ID | Stage G1: >=90, Stage G2: [60–89], Stage G3a: [45–59], Stage G3b: [30–44], Stage G4: [15–29], Stage G5: <15 |
|  | ACR category (mg/mmol) | Qualitative, time-fixed in the year before ID | A1: <3 (Normal to mildly increased), A2: [3–30] (Moderately increased), A3: >30 (Severely increased) |
|  | Classification of CDK using GFR and ACR categories | Qualitative, time-fixed in the year before ID | no CKD, mild CKD, moderate CKD, severe CKD |
|  | At least one test other than lipids/renal | Qualitative, time-fixed in the year before ID | Yes, no |
| **Health care utilization** | Number of GP consultations | Qualitative, time-fixed in the year before ID | 0, 1-3, 4-6, 7-12, >12 |
|  | Hospitalization cumulative duration (days) | Qualitative, time-fixed in the year before ID | 0, 1, 2-7, >7 |
| **Other** | Prior follow-up duration (years) | Continuous, time-fixed at end of follow-up | - |

Abbreviations: ACR, albumin creatinine ratio; BMI, body mass index; BP, blood pressure; CKD, chronic kidney disease; GFR, glomerular filtration ratio; GP, general practitioner; HDL, high-density lipoprotein; HT, hypertension; ID, index date; IHD, ischemic heart disease; IMD, indices of multiple deprivation; LDL, low-density lipoprotein; PAD, peripheral arterial disease; TIA, transient ischemic attack.

# Figure 1a: Absolute standardized differences of covariates before and after propensity score matching in all hypertensive patients


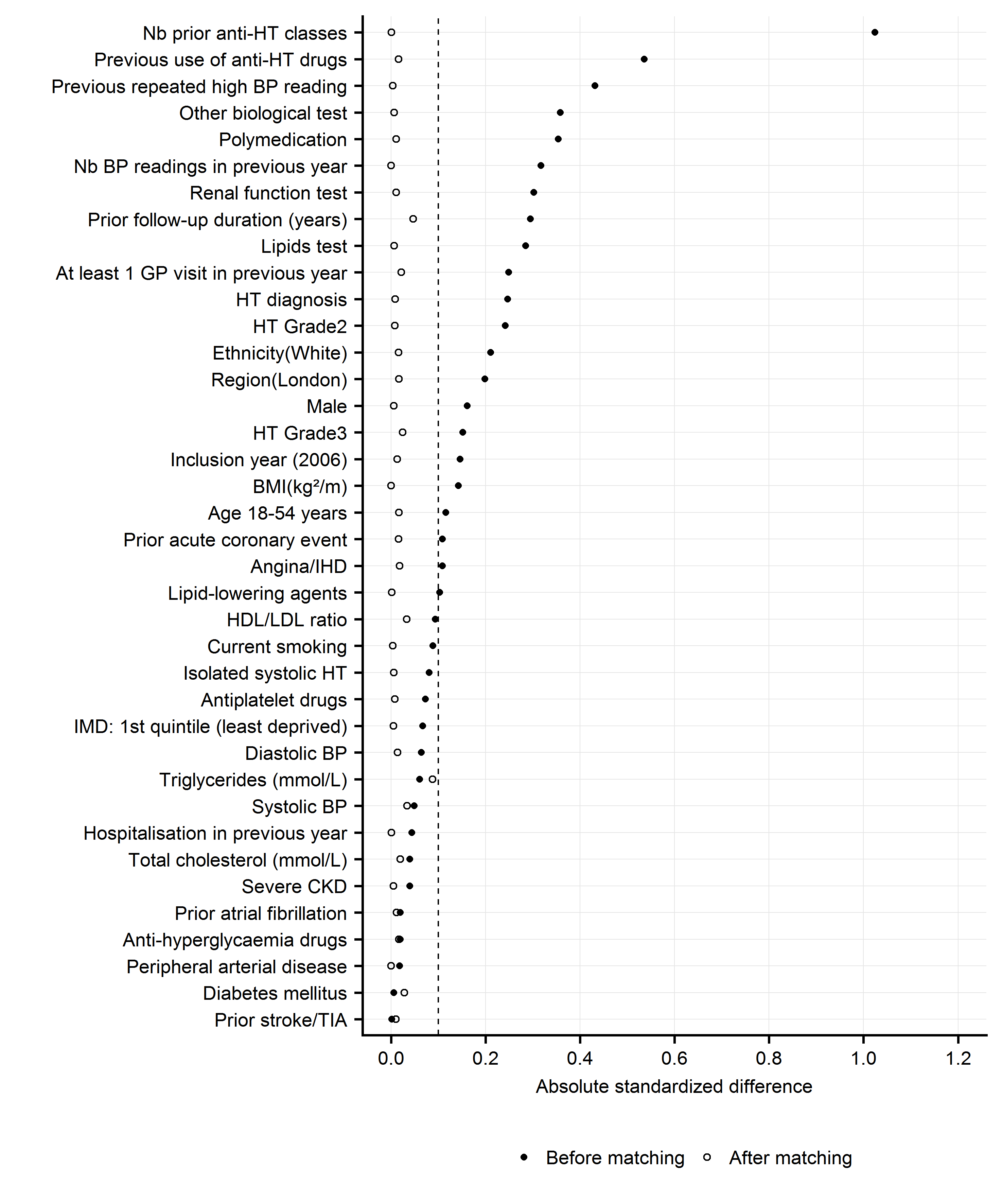


# Figure 1b: Absolute standardized differences of covariates before and after propensity score matching in patients with ACEi and/or CCB


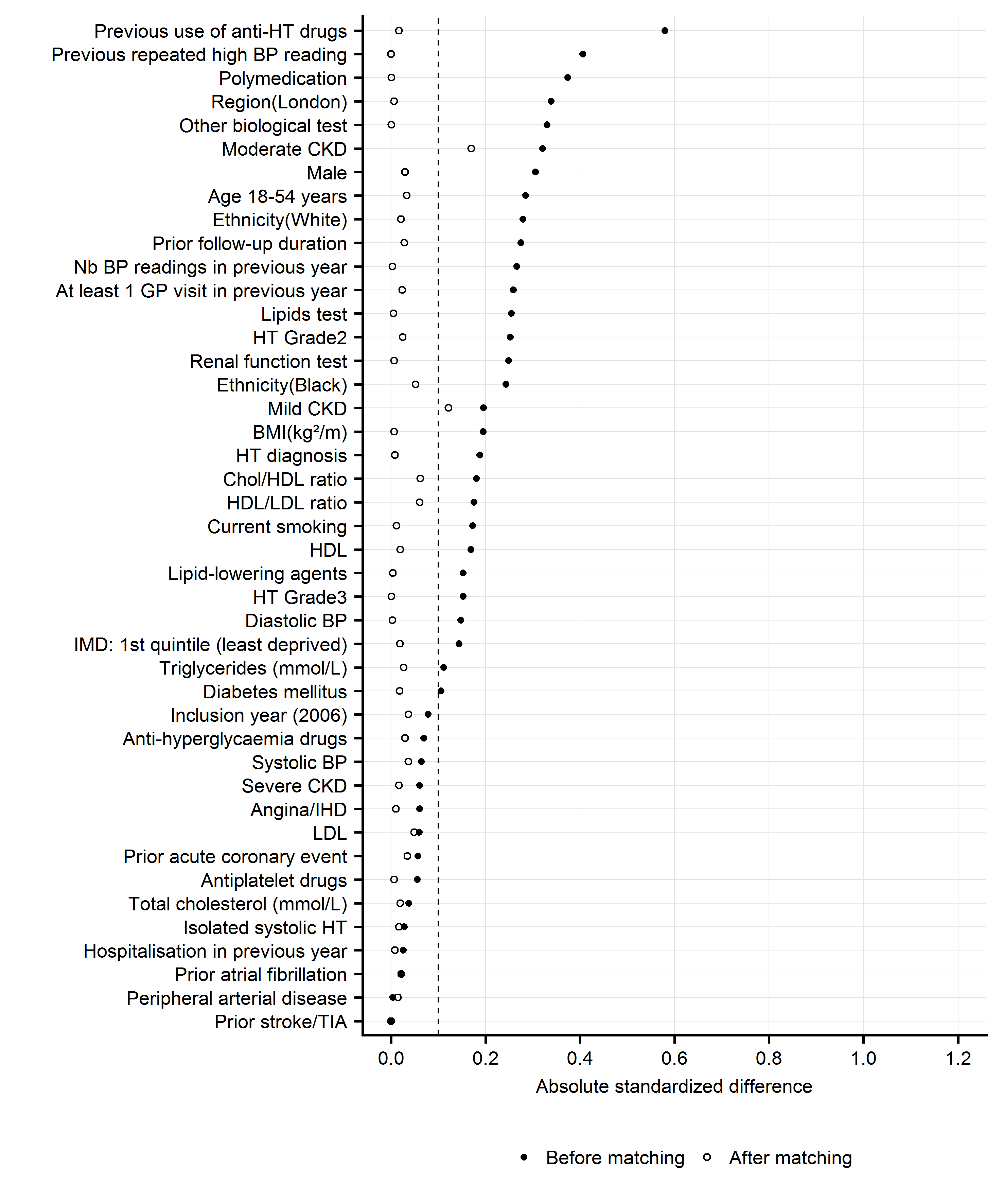


# Figure 1c: Absolute standardized differences of covariates before and after propensity score matching in patients with grade 1 hypertension


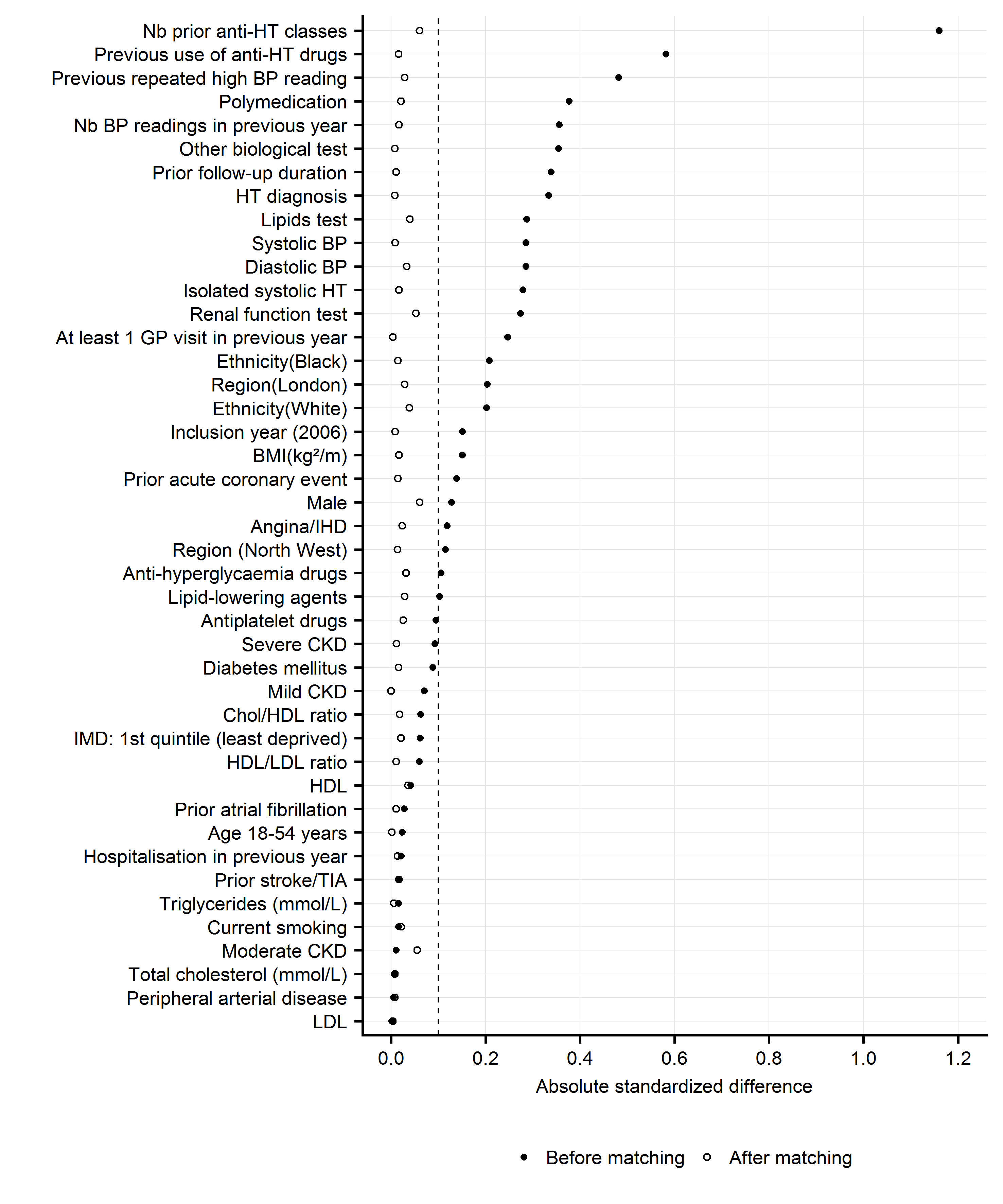


# Figure 1d: Absolute standardized differences of covariates before and after propensity score matching in patients with grade2-3 hypertension


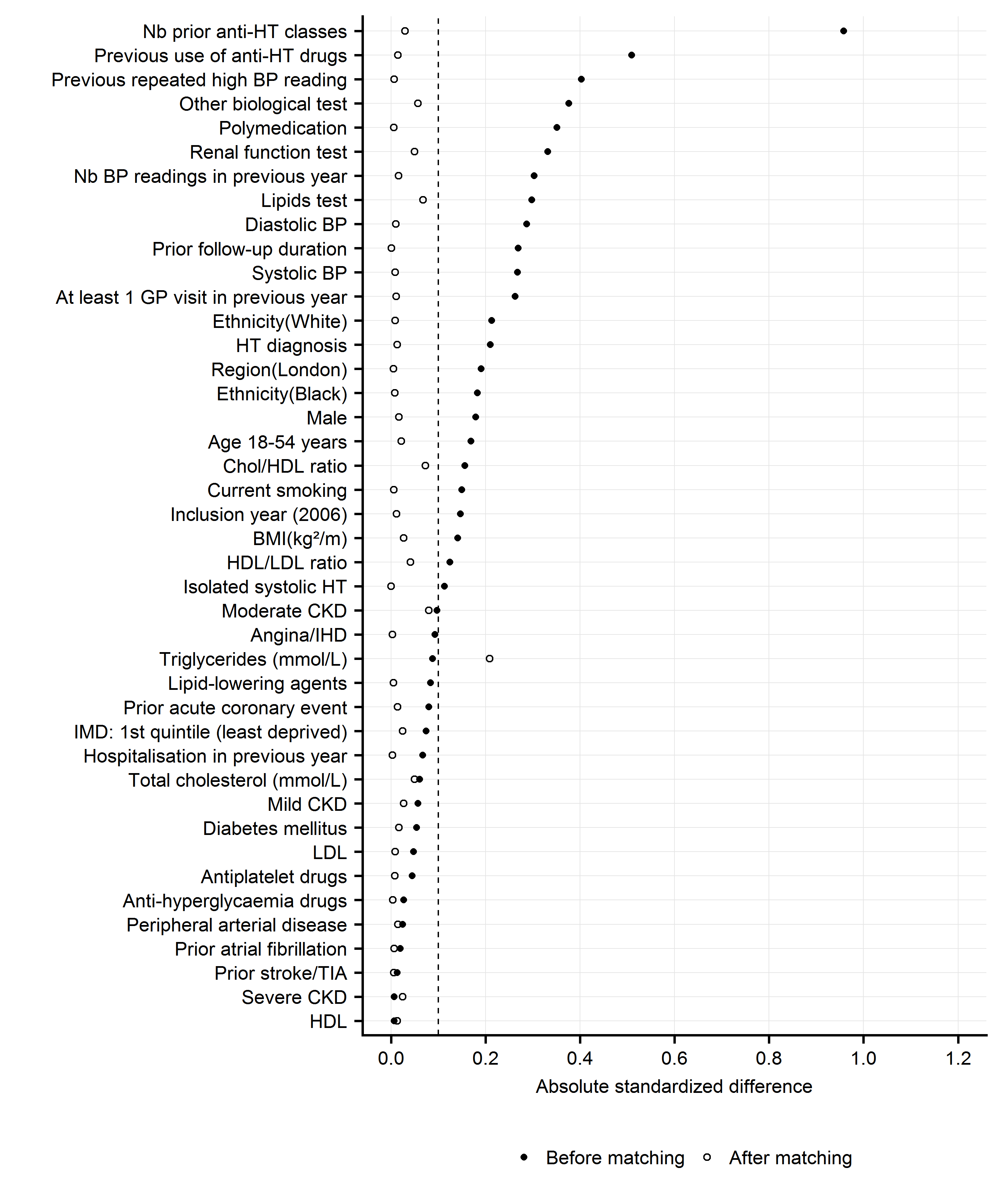


Abbreviations: BMI, body mass index; BP, blood pressure; CKD, chronic kidney disease; GP, general practitioner; HDL, high-density lipoprotein; HT, hypertension; IHD, ischemic heart disease; IMD, indices of multiple deprivation; LDL, low-density lipoprotein; TIA, transient ischemic attack.

# Figure 2: Distribution of propensity score before and after matching


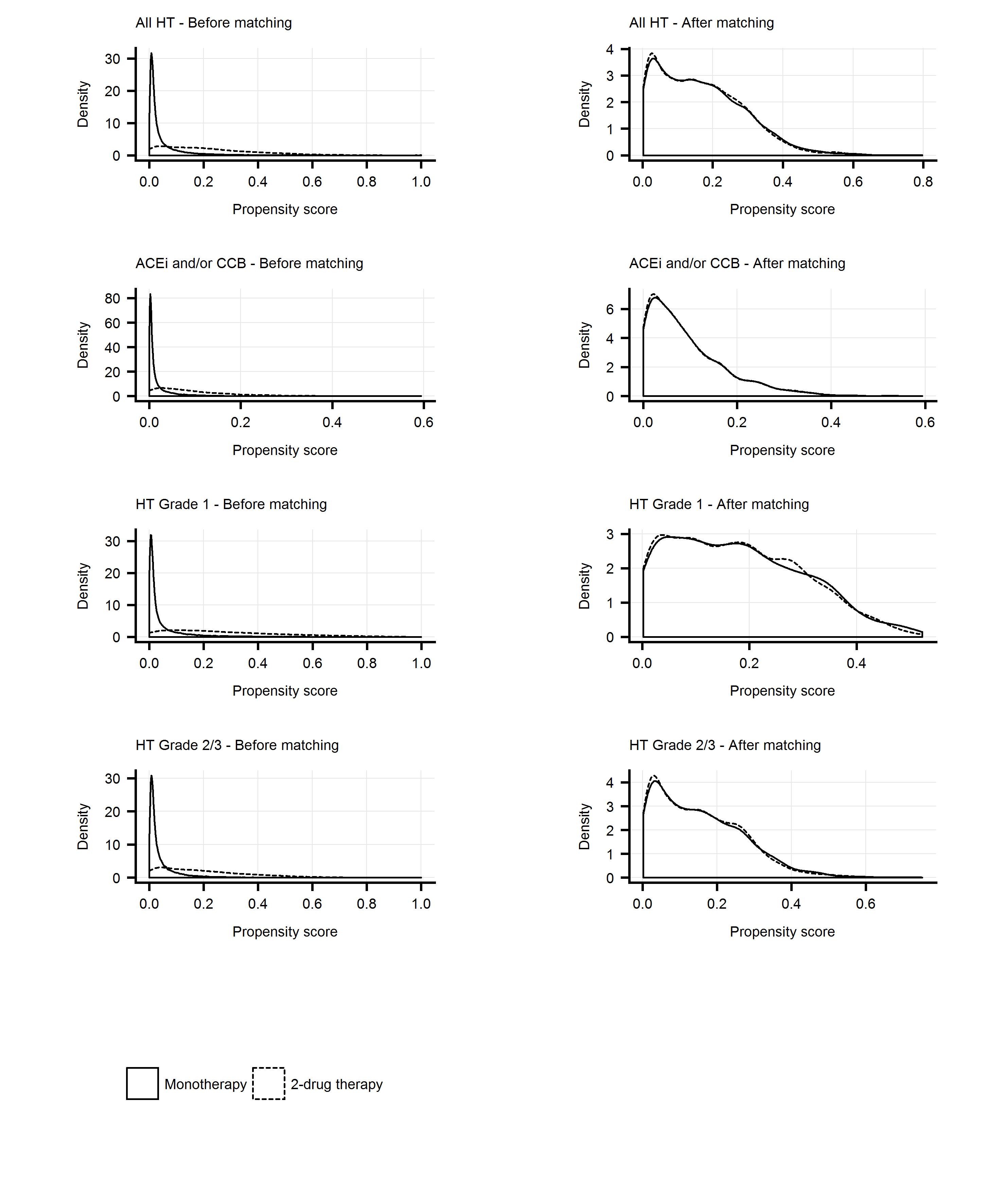
Abbreviations: ACEi, angiotensin-converting enzyme inhibitors; CCB, calcium channel blockers; HT, hypertension.

# Figure 3: Kaplan Meier survival curves for time to achieve blood pressure control


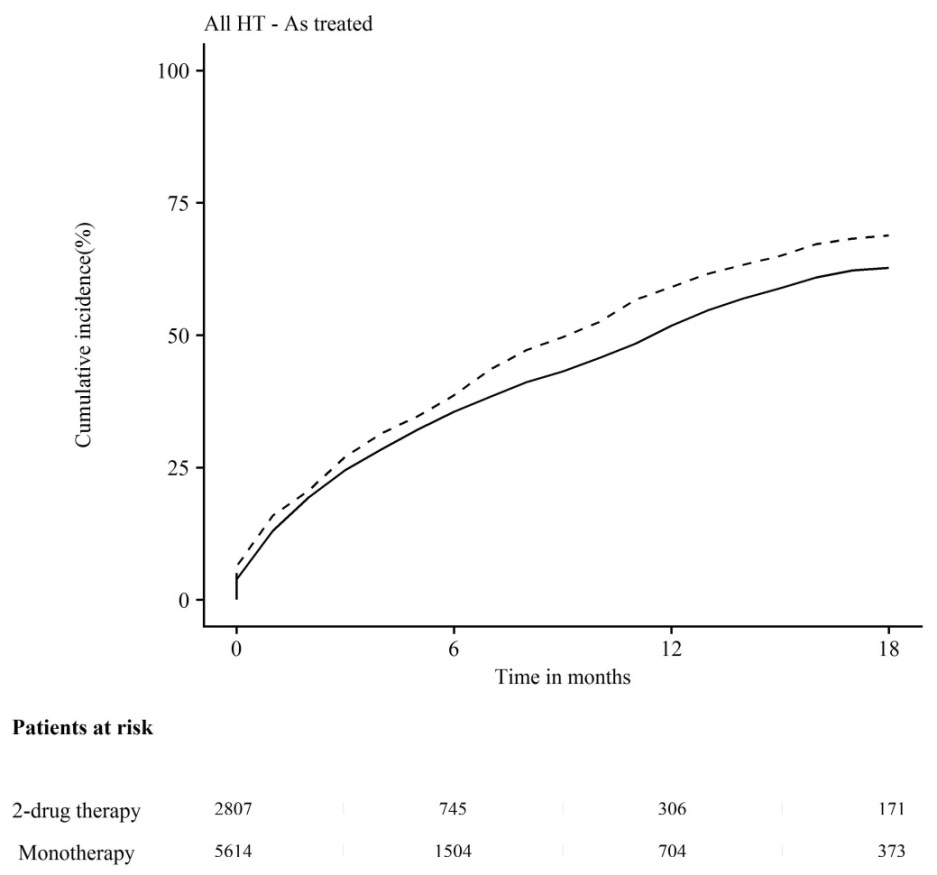


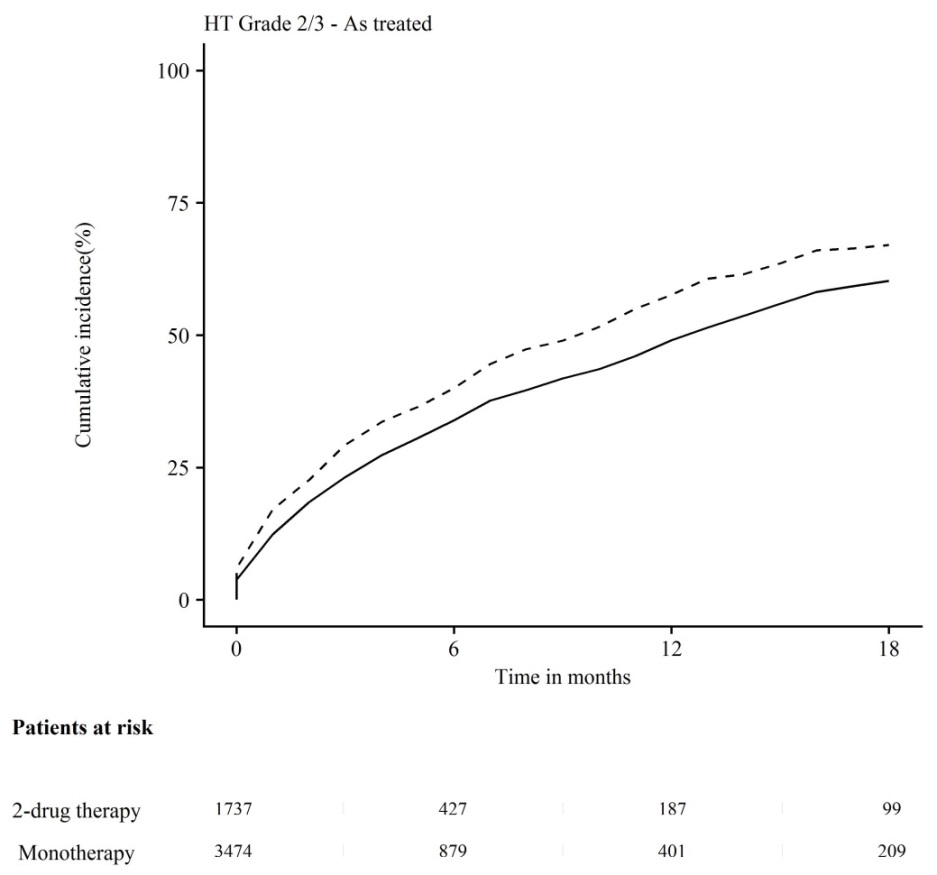


Abbreviations: AT, as treated; HT, hypertension.
